# Identification of Components of the Aggregation Pheromone of the Guam Strain of Coconut Rhinoceros Beetle, *Oryctes rhinoceros,* and Determination of Stereochemistry

**DOI:** 10.1007/s10886-021-01329-z

**Published:** 2021-11-11

**Authors:** David R. Hall, Steven J. Harte, Dudley I. Farman, Mark Ero, Alfred Pokana

**Affiliations:** 1 Natural Resources Institute, University of Greenwich, Central Avenue, Chatham Maritime, Kent, ME4 4TB UK; 2 Papua New Guinea Oil Palm Research Association (PNGOPRA), Dami Research Station, PO Box 97, West New Britain Province, Kimbe, Papua New Guinea; 3 Present Address: Pacific Community (SPC), Land Resources Division, Private Mail Bag, Suva, Fiji; 4 New Britain Palm Oil Limited, Quadalcanal Plains, PO Box 2001, Honiara, Solomon Islands

**Keywords:** (*R*)-4-methyloctanoic acid, Ethyl (*R*)-4-methyloctanoate, Enzymatic resolution, Electroantennogram, Enantioselective gas chromatography

## Abstract

The coconut rhinoceros beetle, *Oryctes rhinoceros* (Linnaeus 1758) (Coleoptera: Scarabaeidae: Dynastinae) (CRB), is endemic to tropical Asia where it damages both coconut and oil palm. A new invasion by CRB occurred on Guam in 2007 and eradication attempts failed using commonly applied *Oryctes rhinoceros* nudivirus (OrNV) isolates. This and subsequent invasive outbreaks were found to have been caused by a previously unrecognized haplotype, CRB-G, which appeared to be tolerant to OrNV. The male-produced aggregation pheromone of the endemic, susceptible strain of *O. rhinoceros* (CRB-S) was previously identified as ethyl 4-methyloctanoate. Following reports from growers that commercial lures containing this compound were not attractive to CRB-G, the aim of this work was to identify the pheromone of CRB-G. Initial collections of volatiles from virgin male and female CRB-G adults from the Solomon Islands failed to show any male- or female-specific compounds as candidate pheromone components. Only after five months were significant quantities of ethyl 4-methyloctanoate and 4-methyloctanoic acid produced by males but not by females. No other male-specific compounds could be detected, in particular methyl 4-methyloctanoate, 4-methyl-1-octanol, or 4-methyl-1-octyl acetate, compounds identified in volatiles from some other species of *Oryctes*. Ethyl 4-methyloctanoate elicited a strong electroantennogram response from both male and female CRB-G, but these other compounds, including 4-methyloctanoic acid, did not. The enantiomers of ethyl 4-methyloctanoate and 4-methyloctanoic acid were conveniently prepared by enzymatic resolution of the commercially-available acid, and the enantiomers of the acid, but not the ester, could be separated by gas chromatography on an enantioselective cyclodextrin phase. Using this approach, both ethyl 4-methyloctanoate and 4-methyloctanoic acid produced by male CRB-G were shown to be exclusively the (*R*)-enantiomers whereas previous reports had suggested male *O. rhinoceros* produced the (*S*)-enantiomers. However, re-examination of the ester and acid produced by male CRB-S from Papua New Guinea showed that these were also the (*R*)-enantiomers. In field trapping experiments carried out in the Solomon Islands, both racemic and ethyl (*R*)-4-methyloctanoate were highly attractive to both male and female CRB-G beetles. The (*S*)-enantiomer and the corresponding acids were only weakly attractive. The addition of racemic 4-methyloctanoic acid to ethyl 4-methyloctanoate did significantly increase attractiveness, but the addition of (*R*)- or (*S*)-4-methyloctanoic acid to the corresponding ethyl esters did not. Possible reasons for the difference in assignment of configuration of the components of the CRB pheromone are discussed along with the practical implications of these results.

**Supplementary Information:**

The online version contains supplementary material available at 10.1007/s10886-021-01329-z.

## Introduction

The coconut rhinoceros beetle, *Oryctes rhinoceros* (Linnaeus 1758) (Coleoptera: Scarabaeidae: Dynastinae) (CRB), is endemic to tropical Asia where it damages both coconut and oil palm, and can kill palms when adults bore into crowns to feed on sap (Bedford, 2013a, 2013b). The beetle was introduced into the Pacific in 1909 via potted soil with rubber tree seedlings from Sri Lanka (Catley, 1969). The pest rapidly multiplied and spread throughout the Pacific Islands. However, it was brought under control by the discovery and distribution of a viral biocontrol agent, *Oryctes rhinoceros* nudivirus (OrNV), previously known as *Rhabdiovirus oryctes* and *Baculovirus oryctes* (Bedford, 2013b; Huger, 2005). Further spread of CRB was suppressed for 30 years (Secretariat of the Pacific Community, 2015) by a strategy of sanitation and biocontrol with OrNV (Jackson, 2009).

However, a new invasion by CRB occurred on Guam in 2007 (Moore et al. 2015) and eradication attempts failed using commonly applied OrNV isolates that cause disease in endemic, susceptible *O. rhinoceros* populations (CRB-S). Subsequently, new CRB invasions have been reported in Port Moresby (Papua New Guinea, 2009), O’ahu (Hawai’i, USA, 2013), and Honiara (Solomon Islands, 2015). Marshall et al. (2017) reported that all of these outbreaks have been caused by a previously unrecognized haplotype, CRB-G, which appeared to be tolerant to OrNV. PCR analysis showed that OrNV was generally present at high incidence in established populations of CRB-S, but absent from the invasive CRB-G populations (Marshall et al. 2017).

The male-produced aggregation pheromone of *O. rhinoceros* was identified as ethyl 4-methyloctanoate by Hallett et al. (1995) using insects from Indonesia. The stereochemistry of the material produced by the beetles was not determined, but, in field trapping tests, traps baited with the racemic or the (*S*)-enantiomer caught similar numbers of beetles and significantly more than those baited with the (*R*)-enantiomer. It has thus generally been assumed that the beetles produce the (*S*)-enantiomer. Hallett et al. (1995) also detected 4-methyloctanoic acid and ethyl 4-methylheptanoate in volatiles from male *O. rhinoceros,* but could not demonstrate any effect of these compounds in field trapping tests. Morin et al. (1996) also found both ethyl 4-methyloctanoate and 4-methyloctanoic acid in widely different relative amounts in volatiles from male *O. rhinoceros* from various geographic origins.

The pheromone, racemic ethyl 4-methyloctanoate, is readily available and is now an important component of IPM strategies against CRB as well as a tool for ecological studies (reviewed by Bedford 2014). Pheromone-baited traps are used to monitor invasion of new plantings by CRB, and in mass trapping to reduce build-up of populations in oil palm breeding sites (Bedford 2014; Maruthadurai and Ramesh 2020; Norman et al. 2007).

However, one of us (ME) received reports from growers in the Port Moresby area of Papua New Guinea, where the CRB-G haplotype is dominant, that pheromone traps baited with commercial lures were catching fewer beetles than expected. If the pheromone of the new, resistant CRB-G haplotype was different from that of the susceptible CRB-S haplotype, this could result in monitoring and control of the pest with the current lures being less effective. Moreover, identification of the pheromone of the CRB-G haplotype could provide a convenient means of monitoring the spread of this resistant haplotype. Thus the aim of this study was to identify components of the aggregation pheromone produced by male CRB-G and to compare these with those produced by the CRB-S strain. This was done by identifying male-specific compounds produced by beetles of the two haplotypes and bioassay of the compounds by electrophysiology and field trapping tests.

Very recently, Etebari et al. (2021) found three major mitochondrial haplotype groups of *O. rhinoceros* across the South Pacific region. Haplotype diversity varied between and within countries and a high incidence of OrNV infection was detected in all haplotypes wherever they occurred, in contrast to the findings of Marshall et al. (2017). However, the original rationale for this work remains valid and the designation of haplotypes used by Marshall et al. (2017) and subsequently by Reil et al. (2018) will be used, i.e. CRB-G for the Guam haplotype tolerant towards OrNV under field conditions and CRB-S for the susceptible haplotype.

## Methods and Materials

### Insect Material

Insects of the Guam strain of *O. rhinoceros* (CRB-G) were collected as pupae in plantations of the Guadalcanal Plains Palm Oil Limited (GPPOL; now part of Sime Darby Group of Companies) in the Solomon Islands during 2016. Marshall et al. (2017) had previously confirmed that CRB from this area were of the Guam strain. Insects of the susceptible strain (CRB-S) were collected from Numondo plantation in West New Britain Province, Papua New Guinea in 2018. Pupae were sent in individual containers to the Natural Resources Institute (NRI), UK, by air freight.

At NRI, pupae were maintained in a quarantine-controlled insectary at 25 °C, 55% RH and 12:12 h L:D cycle. On emergence, adults were separated by sex according to Mini and Prabhu (1988): males have a larger horn and bare pygidium compared with the bushy pygidium with densely packed hairs of females. Adults were maintained under the above conditions on sterilized soil with banana as food.

### Collection of Volatiles

Volatiles were collected from 1 to 7 individuals. Most collections were made from individuals of one sex, but some were made from both sexes together. These were placed in a 5-l round-bottomed, bolt-head flask with shredded paper and a piece of sugarcane. Some collections were made with banana or tinned palm hearts as food, but these produced much larger amounts of volatiles than sugarcane. Air was drawn into the flask (2 l/min) through a filter containing activated charcoal (20 cm × 1.5 cm; 6-10 mesh) and out through a filter made from a Pasteur pipette (4 mm i.d.) containing Porapak Q (200 mg; 50-80 mesh; Supelco, Gillingham, Dorset, UK). The Porapak Q was extracted with chloroform in a Soxhlet apparatus for 6 h and washed with dichloromethane immediately before use. Collections were made for periods from 24 h – 72 h. Trapped volatiles were removed with dichloromethane (Pesticide Residue Grade; 1 ml) and stored at 4 °C before analysis.

To convert esters in the collections of volatiles to the corresponding acids, 0.5 ml of a collection containing approximately 50 μg ethyl 4-methyloctanoate was evaporated just to dryness under a gentle stream of nitrogen. Ethanol (50 μl) and 2 N aqueous potassium hydroxide solution (50 μl) were added and the mixture left for 6 h at room temperature. Water (0.5 ml) was added and the mixture was extracted with hexane (0.5 ml). After acidification with aqueous 4 N sulfuric acid (50 μl), the aqueous layer was extracted with two portions of diethyl ether (0.5 ml) and the combined extracts dried with anhydrous magnesium sulfate.

### Analysis by Gas Chromatography

Collections of volatiles were analyzed by gas chromatography (GC) with flame ionization detection (FID) using HP6850 instruments (Agilent Technologies, Stockport, Cheshire, UK) fitted with a fused silica column (30 m × 0.32 mm i.d. 0.25 μm film thickness) coated with non-polar HP5 (Agilent) or polar DBWax (Supelco). The oven temperature was held at 50 °C for 2 min then programmed at 10 °C/min to 250 °C and held for 5 min. Carrier gas was helium (2.4 ml/min), injection was splitless (220 °C) and detection by FID (250 °C).

Enantioselective gas chromatography was carried out on a CP-Chirasil-Dex CB column (25 m × 0.32 mm; 0.25 μm film thickness; Varian/Agilent) with helium carrier gas (2.4 ml/min), split injection (220 °C; 20:1) and FID (220 °C). The oven temperature was held at 60 °C for 2 min then programmed at 5 °C/min to 200 °C.

Collections were also analyzed by GC coupled to mass spectrometry (MS) using a Varian 3700 GC linked directly to a Saturn 2200 ion-trap MS (Varian, now Agilent). Columns (30 m × 0.25 mm i.d. 0.25 μm film thickness) were coated with polar DBWax (Supelco) or non-polar VF5 (Varian/Agilent). The carrier gas was helium (1 ml/min) and the oven temperature was held at 40 °C for 2 min then programmed at 10 °C/min to 250 °C and held for 5 min.

Retention Indices for compounds were calculated relative to the retention times of *n*-alkanes. Amounts present in collections were estimated by comparison of peak areas with those of external standards. The response factor for 4-methyloctanoic acid was much lower than that of the corresponding ester in both GC-FID and GC-MS.

### Analysis by Gas Chromatography Coupled to Electroantennographic Recording (GC-EAG)

GC-EAG Analyses were carried out on a HP6890 GC (Agilent) fitted with flame ionization detector (FID) and fused silica capillary columns (30 m × 0.32 mm i.d. × 0.25 μm film thickness) coated with DBWax and DB5 (Supelco). Injections onto the DBWax column were in splitless mode (220 °C), carrier gas was helium (2.4 ml/min) and the oven temperature was held at 50 °C for 2 min and then programmed at 10 °C/min or 20 °C/min. to 250 °C and held for 3 min. The effluents of the two columns were combined with a glass push-fit Y-tube connector (Agilent) connected to a second Y-tube connector with deactivated fused silica tubing (10 cm × 0.32 mm i.d.). One arm of this connector was connected with deactivated fused silica tubing (50 cm × 0.32 mm i.d.) to the FID (250 °C) and the other to an equal length of deactivated silica tubing passing through a heated transfer line (250 °C; Syntech, Hilversum, The Netherlands, now Kirchzarten, Germany) into a glass tube (4 mm i.d.) through which air passed (500 ml/min) over the EAG preparation. Both the FID and EAG signals were collected and analyzed with EZChrom software (Elite v3.0; Agilent Technologies).

EAG preparations were made with glass microelectrodes filled with saline (0.1 M KCl with 1% polyvinylpyrrolidone) attached to silver wire electrodes held by integrated electrode holders, micromanipulators and amplifier (INR-2; Syntech). One antenna was removed and the base inserted into the indifferent electrode. Following the description of the antennal morphology on *O. rhinoceros* (Renou et al., 1998), the club was opened out to reveal the lamellar structure and the end of the recording electrode was placed on the surface of one of the lamellae. EAG responses were amplified ×10.

### Chemicals

Racemic ethyl 4-methyloctanoate was prepared by heating commercially available 4-methyloctanoic acid (SigmaAldrich, Gillingham, Dorset, UK) in ethanol with a drop of sulfuric acid for 1 h at reflux, followed by evaporation of most of the ethanol. This was repeated with a further portion of ethanol, followed by aqueous work-up and fractional distillation to give the product in 86% yield.

The enantiomers of 4-methyloctanoic acid and the corresponding ethyl ester were conveniently resolved enzymatically in multigram quantities as reference standards and for field testing. Heinsman et al. (1998, 2001) reported that immobilized lipase from *Candida antarctica* acted selectively on the (*R*)-enantiomer*,* so that the acid could be selectively esterified in ethanol or the ethyl ester could be selectively hydrolyzed in 1 M aqueous K_2_HPO_4_. Moreover, the acid could be separated from the ester by extraction with aqueous base and subsequent acidification without resort to chromatography.

**Fig. 1:**
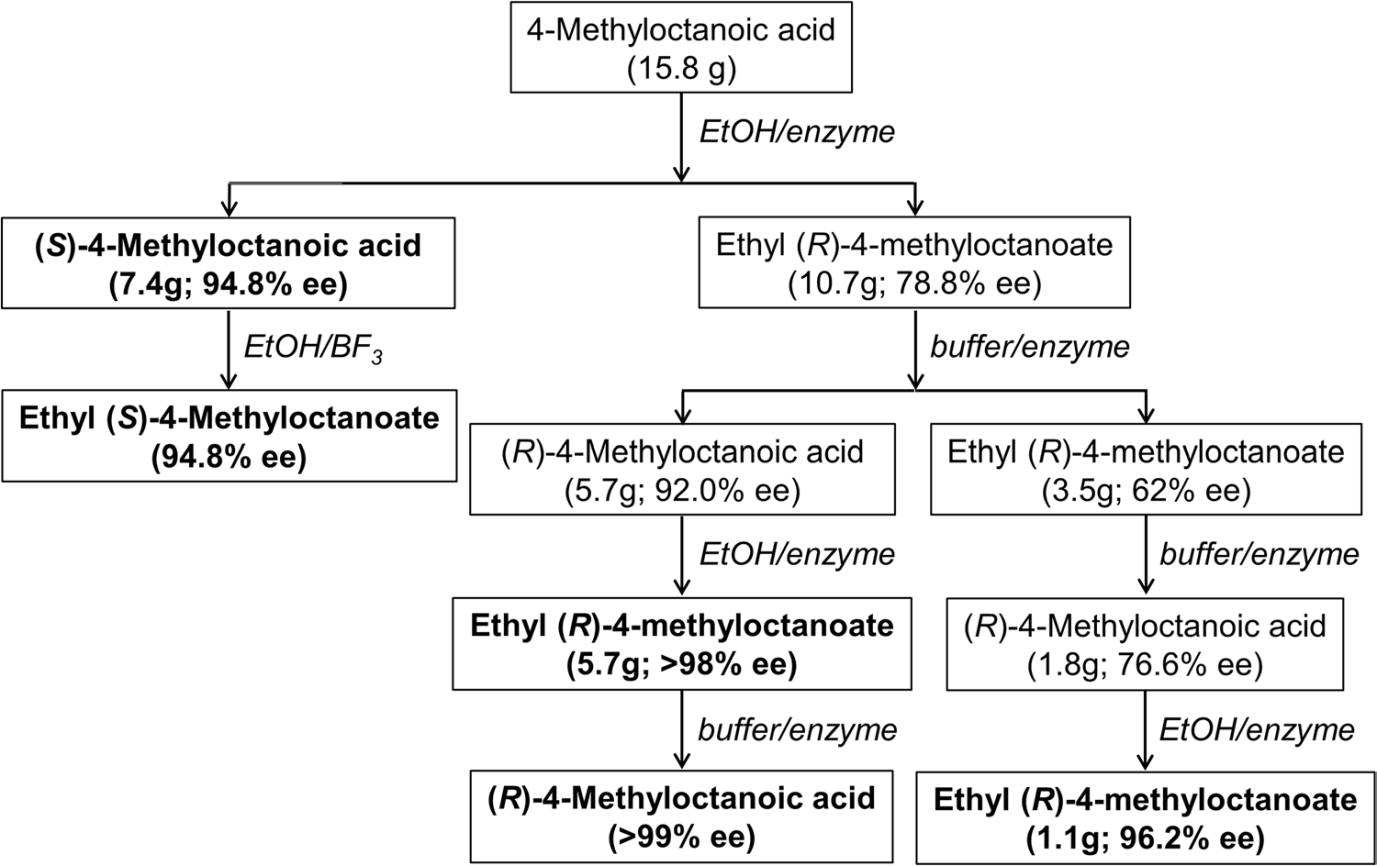
Enzymatic resolution of racemic 4-methyloctanoic acid (enzyme: immobilized lipase from *Candida antarctica*; buffer: aqueous 1 M K_2_HPO_4_; samples in bold were used in field trapping experiments)

The sequence of reactions is shown in Fig. 1 with the enantiomeric excess (ee) of the products, and detailed procedures are described in the Supplementary Material. Enantiomers of ethyl 4-methyloctanoate could not be separated by GC analysis on the cyclodextrin column used here, but those of the corresponding acid could, as also found by Karl et al. (1994b) (ester 12.10 min, (*R*)-acid 18.06 min, (*S*)-acid 18.14 min). Thus the enantiomeric composition of the ester products was assessed by hydrolyzing a small portion of the ester with potassium hydroxide in aqueous ethanol for 6 h and work-up as described above for collections of volatiles. The stereochemistry of the products was further confirmed by comparison with a sample of 4-methyloctanoic acid enriched in the (*R*)-enantiomer provided by Prof Angel Guerrero (Muñoz et al. 2009).

Methyl 4-methyloctanoate was prepared by esterification of 4-methyloctanoic acid with methanol, catalyzed by boron trifluoride etherate. 4-Methyloctan-1-ol was prepared by reduction of ethyl 4-methyloctanoate with lithium aluminum hydride in ether and acetylated with acetic anhydride in pyridine to give 4-methyloctyl acetate. Details are given in the Supplementary Material. Compounds were characterized by their ^1^H- and ^13^C-NMR spectra, their GC retention indices (Supplementary Material Table S2) and mass spectra (Supplementary Material Figs. S1, S2).

(*E*)-Geranic acid was prepared by low temperature crystallization (−80 °C) of commercially available geranic acid (SigmaAldrich) in petroleum spirit (b.p. 40-60 °C) or acetone. Analysis of the commercial material on a polar GC column showed the (*E*)- and (*Z*)-isomers in 76:24 ratio. Two recrystallizations gave material >99% (*E*)-isomer.

### Pheromone Dispensers

Sealed polyethylene vials (30 mm × 15 mm × 1.5 mm thick; Fisher Scientific, Loughborough, Leicestershire, UK) and polyethylene sachets (5 cm × 5 cm × 60 μm thick, 5 cm × 5 cm × 120 μm, 2.5 cm × 5 cm × 250 μm) prepared by heat sealing layflat tubing (Transpack Ltd., Southampton, UK) were evaluated as dispensers in laboratory and field. The test compound was impregnated onto cotton dental rolls (19 mm × 8 mm and 38 mm × 8 mm in vials and sachets, respectively). Standard CRB lures were blister packs (27 mm diameter) containing approximately 1 ml ethyl 4-methyloctanoate (P046 Sime RB Pheromone, distributed by Sime Darby Agri-Bio Sdn Bhd, Selangor, Malaysia, and manufactured by ChemTica Internacional, Costa Rica).

Release rates were determined for dispensers maintained in a laboratory fume hood at 20-22 °C by periodic weighing of duplicate dispensers and calculation of mean weight loss. Release rates are given in Table 1 and data are shown in Supplementary Material Figs. S4-S7).

**Table 1:** Release rates of ethyl 4-methyloctanoate, 4-methyloctanoic acid and geranic acid from dispensers measured under laboratory conditions by weight loss (*N* = 2; 20-22 °C; for data see Supplementary Material Figs. S4-S7)

	Mean release rate (mg/d)				
		Polyethylene sachet			
Compound	Polyethylene vial	5 cm × 5 cm × 60 μm	5 cm × 5 cm × 120 μm	2.5 cm × 5 cm × 250 μm	Standard ChemTica
Ethyl 4-methyloctanoate	1.0			20.0	9.1
4-Methyloctanoic acid		1.5	1.4	1.2	
Geranic acid		0.6	0.3		

### Field Trapping Experiments

#### Traps

Traps were those used as standard in the Solomon Islands, constructed from PVC pipe (3 m long × 15 cm diameter x approx. 10 mm thick) with open ends positioned vertically with the lower end in a bucket on the ground to capture beetles (Supplementary Material Fig. S3). Beetles could enter at the top end and by two windows (20 cm × 10 cm) cut in opposite sides of the pipe at 40 cm and 70 cm from the top respectively. The lure was hung inside the pipe 20 cm from the top end. The bucket was half-filled with organic debris to retain trapped beetles.

#### Trapping Experiments

Two field trapping experiments were carried out in plantations of the Guadalcanal Plains Palm Oil Limited (GPPOL; now part of Sime Darby Group of Companies) in the Solomon Islands. Marshall et al. (2017) had previously confirmed that CRB from this area were of the Guam strain. In the first experiment (8 May – 6 June 2017), catches were compared in traps baited with single components, racemic, (*S*)- and (*R*)-enantiomers of ethyl 4-methyloctanoate dispensed from polyethylene vials, racemic, (*S*)- and (*R*)-enantiomers of 4-methyloctanoic acid dispensed from a standard thickness (120 μm) sachet and (*E*)-geranic acid in a thin-walled sachet (60 μm).

In the second experiment (20 June – 11 July 2017), catches were compared in traps baited with racemic, (*S*)- and (*R*)-enantiomers of ethyl 4-methyloctanoate dispensed from polyethylene vials, combinations of racemic, (*S*)- and (*R*)-enantiomers of ethyl 4-methyloctanoate with the corresponding acid dispensed from polyethylene vials and thick (250 μm) polyethylene sachets respectively, and ethyl 4-methyloctanoate dispensed at a higher rate from a sachet. Both experiments included unbaited traps and traps baited with the standard ChemTica lure as negative and positive controls respectively. Details of the lures are given in Table 2.

**Table 2:** Dispensers and loadings of lures used in two field trapping experiments

	Dispenser/Amount			
		Polyethylene sachet		
Compound	Polyethylene vial	5 cm × 5 cm × 60 μ	5 cm × 5 cm × 120 μ	2.5 cm × 5 cm × 250 μ
Experiment 1				
Ethyl 4-methyloctanoate	100 μl			
Ethyl (*S*)-4-methyloctanoate	100 μl			
Ethyl (*R*)-4-methyloctanoate	100 μl			
4-Methyloctanoic acid			100 μl	
(*S*)-4-Methyloctanoic acid			100 μl	
(*R*) 4-Methyloctanoic acid			100 μl	
Geranic acid		100 μl		
Experiment 2				
Ethyl 4-methyloctanoate	100 μl			
Ethyl (*S*)-4-methyloctanoate	100 μl			
Ethyl (*R*)-4-methyloctanoate	100 μl			
Ethyl 4-methyloctanoate +4-methyloctanoic acid	100 μl			100 μl
Ethyl (*S*)-4-methyloctanoate + (*S*)-4-methyloctanoic acid	100 μl			100 μl
Ethyl (*R*)-4-methyloctanoate + (*R*)-4-methyloctanoic acid	100 μl			100 μl
Ethyl 4-methyloctanoate				500 μl

Traps were deployed in randomized complete blocks with 50 m between traps in a block and at least 100 m between blocks. There were six replicate blocks in each experiment and catches were sexed, counted and discarded weekly. Total catches were transformed to log(x + 1) and subjected to analysis of variance, omitting treatments with zero catches. Where significant differences (*P* < 0.05) were indicated, differences between means were tested for significance (*P* < 0.05) by a Least Significant Difference (LSD) test.

## Results

### Collection and Analysis of Pheromone of Guam Strain of *Oryctes rhinoceros* (CRB-G)

**Fig. 2:**
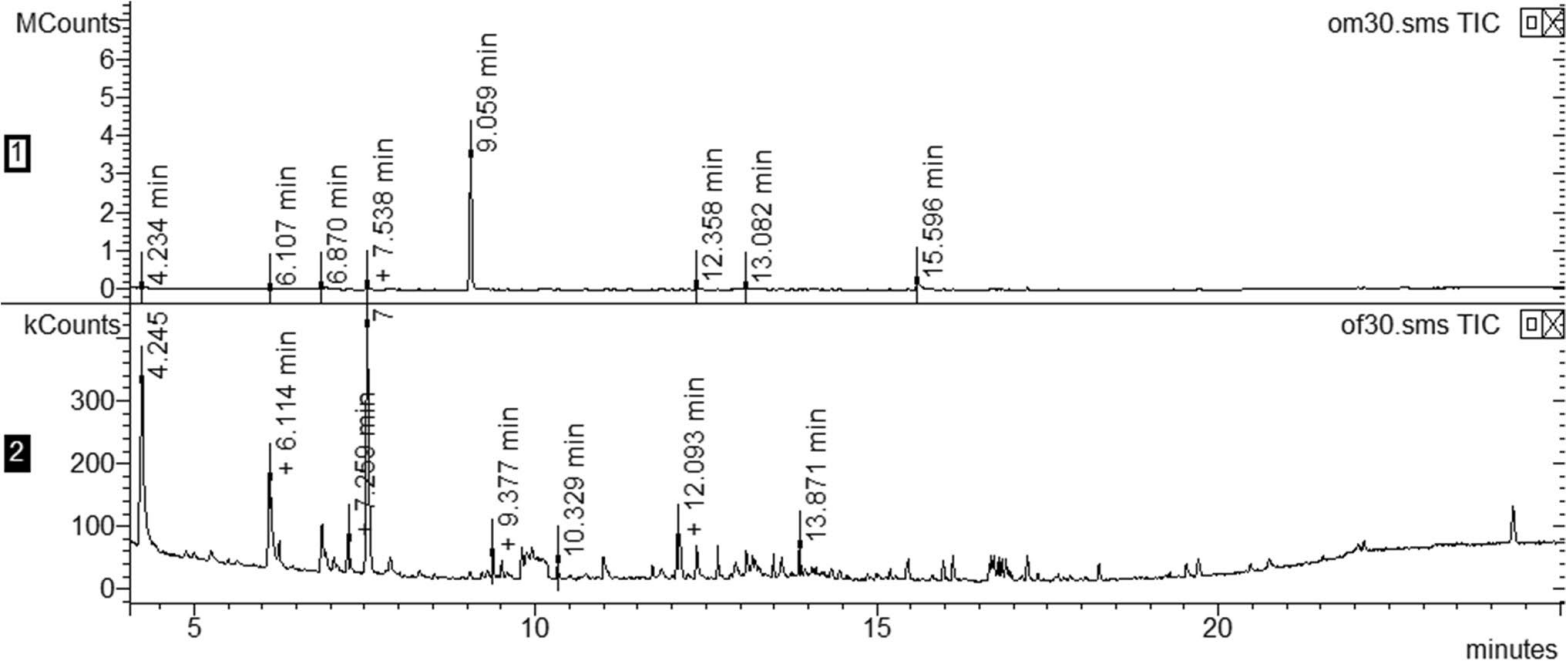
GC-MS analyses of volatiles collected from five male Guam strain *Oryctes rhinoceros* (CRB-G) (upper) and seven female CRB-G (lower); ethyl 4-methyloctanoate at 9.06 min, 4-methyloctanoic acid at 15.60 min

CRB-G insects were received at NRI on 4 November 2016 and either emerged in transit or soon after arrival. Collections of volatiles were made at least once a week from male and female beetles separately (*N* = 32 collections each) through until 18 April 2017. No obvious differences in volatiles collected from males and females were detected by GC-MS analyses until 12 April 2017, i.e. over 5 months after emergence, when ethyl 4-methyloctanoate was detected. A collection from five males for 24 h yielded approximately 100 μg ethyl 4-methyloctanoate and 25 μg 4-methyloctanoic acid, while neither compound could be detected in a parallel collection from seven females (Fig. 2). Subsequent collections from the same males yielded amounts of the ester and acid, respectively, of 200 μg and 400 μg from three males and 250 μg and 600 μg from one male, both for 65 h, followed by 20 μg acid only from two individual males for 48 h, and finally 7.5 μg acid from the one remaining male over 24 h.

The only exception to the above was in early December 2016 when the (*E*)- and (*Z*)-isomers of geranic acid were detected in 97:3 ratio. In collections from a single male for 60 h 850 μg was detected, followed by 180 μg during the next 24 h. Parallel collections from a single female yielded 100 μg and 20 μg respectively. Subsequent collections from different insects on fresh sugarcane yielded small but detectable amounts of geranic acid.

The identifications of ethyl 4-methyloctanoate and 4-methyloctanoic acid were confirmed by identical retention times with synthetic standards in GC-FID analyses on polar, non-polar and cyclodextrin GC columns, and by identical retention times and mass spectra in GC-MS analyses on a polar GC column. Methyl 4-methyloctanoate, 4-methyl-1-octanol and the corresponding acetate could not be detected.

**Fig. 3:**
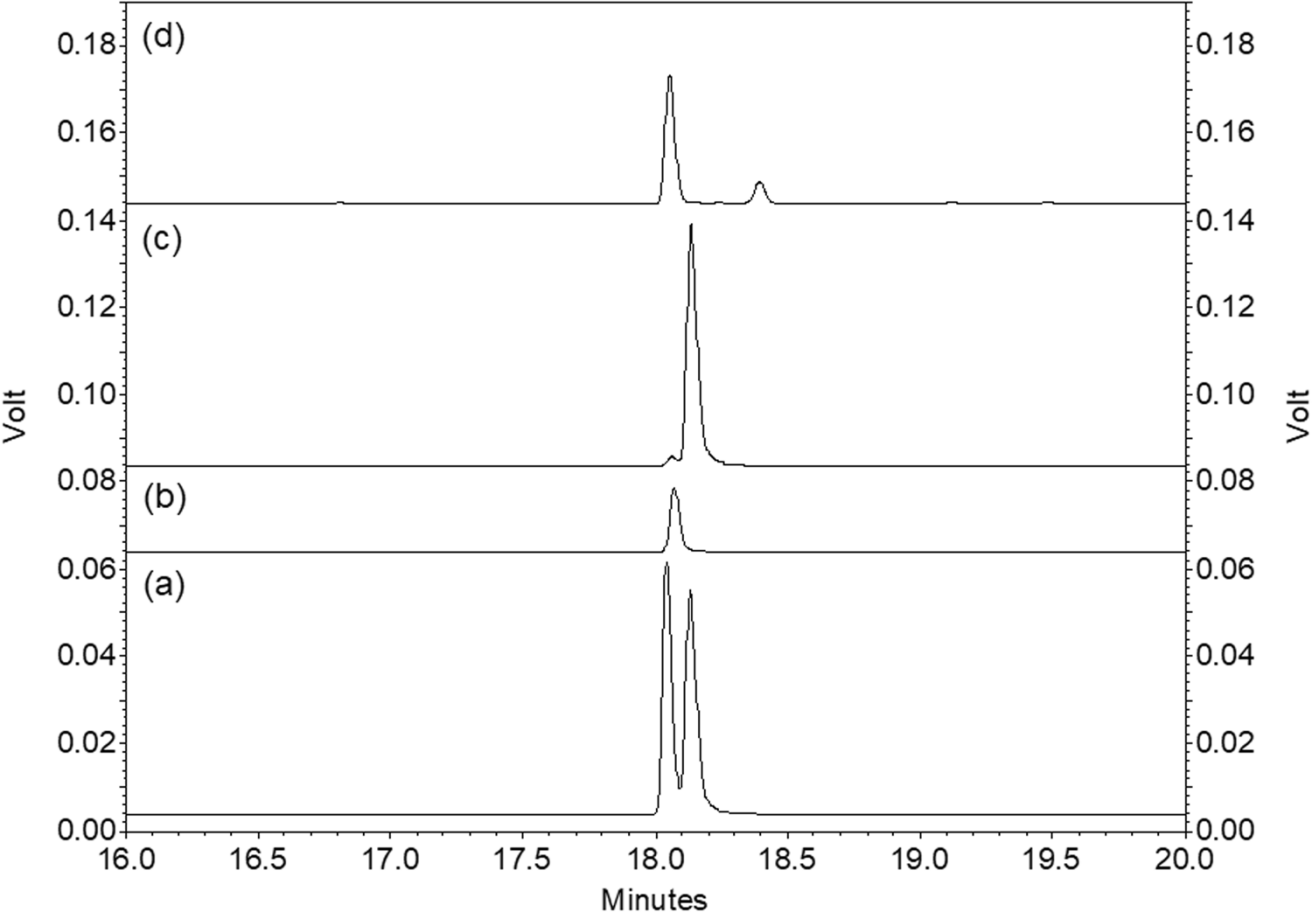
Analyses on enantioselective cyclodextrin GC column of (from bottom) (a) racemic 4-methyloctanoic acid, (b) (*R*)-4-methyloctanoic acid, (c) (*S*)-4-methyloctanoic acid, and (d) a volatile collection from male Guam strain *Oryctes rhinoceros* beetles (CRB-G) after hydrolysis of ethyl 4-methyloctanoate to 4-methyloctanoic acid, showing this is exclusively the (*R*)-enantiomer

In analyses of volatile collections containing both ethyl 4-methyloctanoate and 4-methyloctanoic acid on the cyclodextrin column, the acid was exclusively the earlier eluting (*R*)-enantiomer. After hydrolysis of the ester in the samples, analysis showed only the (*R*)-enantiomer of the acid, confirming that the ester also had the (*R*)-configuration (Fig. 3).

### Analysis by Gas Chromatography Coupled to Electroantennographic Recording (GC-EAG)

In GC-EAG analyses of early collections of volatiles from male or female CRB-G beetles, no consistent EAG responses were detected from antennae of male or female beetles (*N* = 6).

**Fig. 4:**
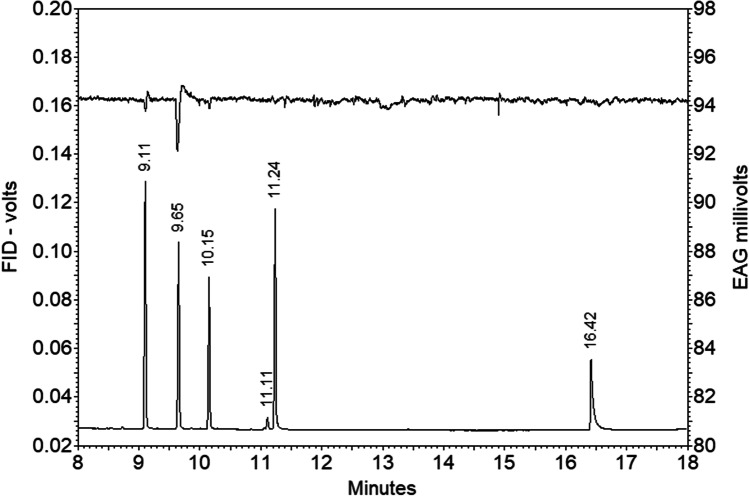
GC-EAG Analysis of synthetic standards with antenna of male Guam strain *Oryctes rhinoceros* (CRB-G). Upper trace EAG, lower trace FID; compounds in order of elution: methyl 4-methyloctanoate, ethyl 4-methyloctanoate, 4-methyl-1-octyl acetate, 4-methyl-1-octanol, 4-methyloctanoic acid

In GC-EAG analyses of synthetic standards, a consistent response was observed from antennae of both males and females to ethyl 4-methyloctanoate. A response was occasionally observed to the corresponding methyl ester, but no response was observed to 4-methyl-1-octanol, the corresponding acetate or 4-methyloctanoic acid (Fig. 4).

EAG responses from antennae of both males and females were generally greater to ethyl (*R*)-4-methyloctanoate than to the (*S*)-enantiomer (Supplementary Material Fig. S7). This was not examined rigorously as available samples of both enantiomers contained small amounts of the opposite enantiomer. However, in one example, mean responses (*N* = 2 delivered alternately) from the antenna of a male beetle were 0.28 mV and 0.14 mV to the (*R*)- and (*S*)-enantiomers (10 ng), respectively, and from the antenna of a female beetle 0.32 mV and 0.10 mV, respectively.

### Pheromone of Susceptible Strain of *Oryctes rhinoceros* (CRB-S)

Beetles and emerging pupae of the susceptible strain of *O. rhinoceros* (CRB-S) were received at NRI on 23 February 2018. Volatiles were collected from males at 3-4 d intervals, but it was not until 10 April 2018 that 4-methyloctanoic acid was detected in collections from males, and ethyl 4-methyloctanoate was not detected until 18 April 2018 (Supplementary Fig. S9). Production of both continued at declining levels until 27 April 2018 after which neither compound was detected until collections were terminated on 16 May 2018. The two collections with most material contained 170 μg and 23 μg ethyl 4-methyloctanoate, and 830 μg and 109 μg 4-methyloctanoic acid, respectively, collected over 24 h from two male beetles. These compounds were never detected in collections from females.

In analyses of volatile collections on the cyclodextrin column, 4-methyloctanoic acid from these beetles co-chromatographed with the (*R*)-enantiomer. After hydrolysis of the ethyl ester present, the analysis showed only (*R*)-4-methyloctanoic acid, confirming that the ester also had the (*R*)-configuration (Supplementary Material Fig. S10).

### Trapping Experiments

**Fig. 5:**
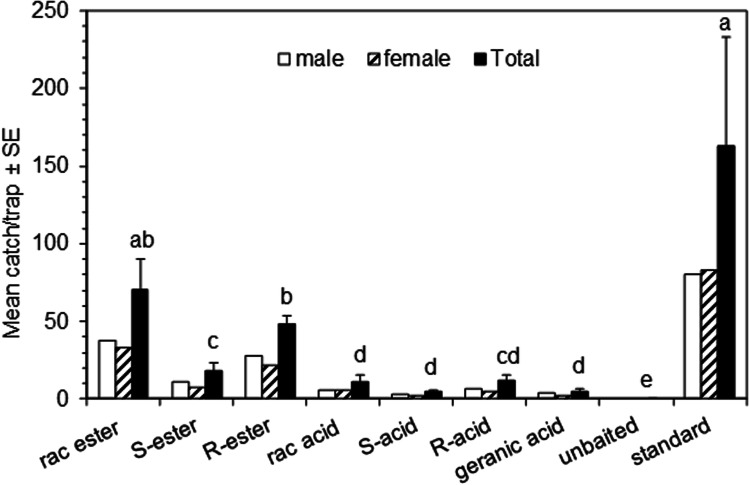
Mean catches of Guam strain *Oryctes rhinoceros* (CRB-G) beetles in traps baited with individual chemicals in Experiment 1 (Solomon Islands; 8 May – 6 June 2017; *N* = 6). Means with different letters are significantly different (*P* < 0.05) after analysis of variance on total catches transformed to log(x + 1) and LSD test of significance of differences between means (*F* = 25.26; df = 7,35; *P* < 0.001). Ester is ethyl 4-methyloctanoate; acid is 4-methyloctanoic acid; geranic acid is (*E*)-geranic acid; standard is Sime Darby/ChemTica lure

In both trapping experiments, catches of male and female *O. rhinoceros* Guam strain beetles (CRB-G) were similar and analyses were carried out on the combined catch. In the first trapping experiment (Fig. 5), catches of CRB-G beetles in traps baited with racemic or the (*R*)-enantiomer of ethyl 4-methyloctanoate were significantly higher than those in traps baited with the (*S*)-enantiomer. Catches in traps baited with the corresponding acids and (*E*)-geranic acid were low, although significantly different from the zero catches in unbaited traps. Catches with the (*S*)-enantiomer of 4-methyloctanoic acid were lower than those with the (*R*)-enantiomer, although not significantly so because of the low numbers caught. Catches were highest in traps baited with the ChemTica lure, although not significantly higher than those baited with racemic ethyl 4-methyloctanoate in a polyethylene vial, even though the release rate was almost an order of magnitude greater (9.1 mg/d and 1.0 mg/d, respectively, Table 1).

**Fig. 6:**
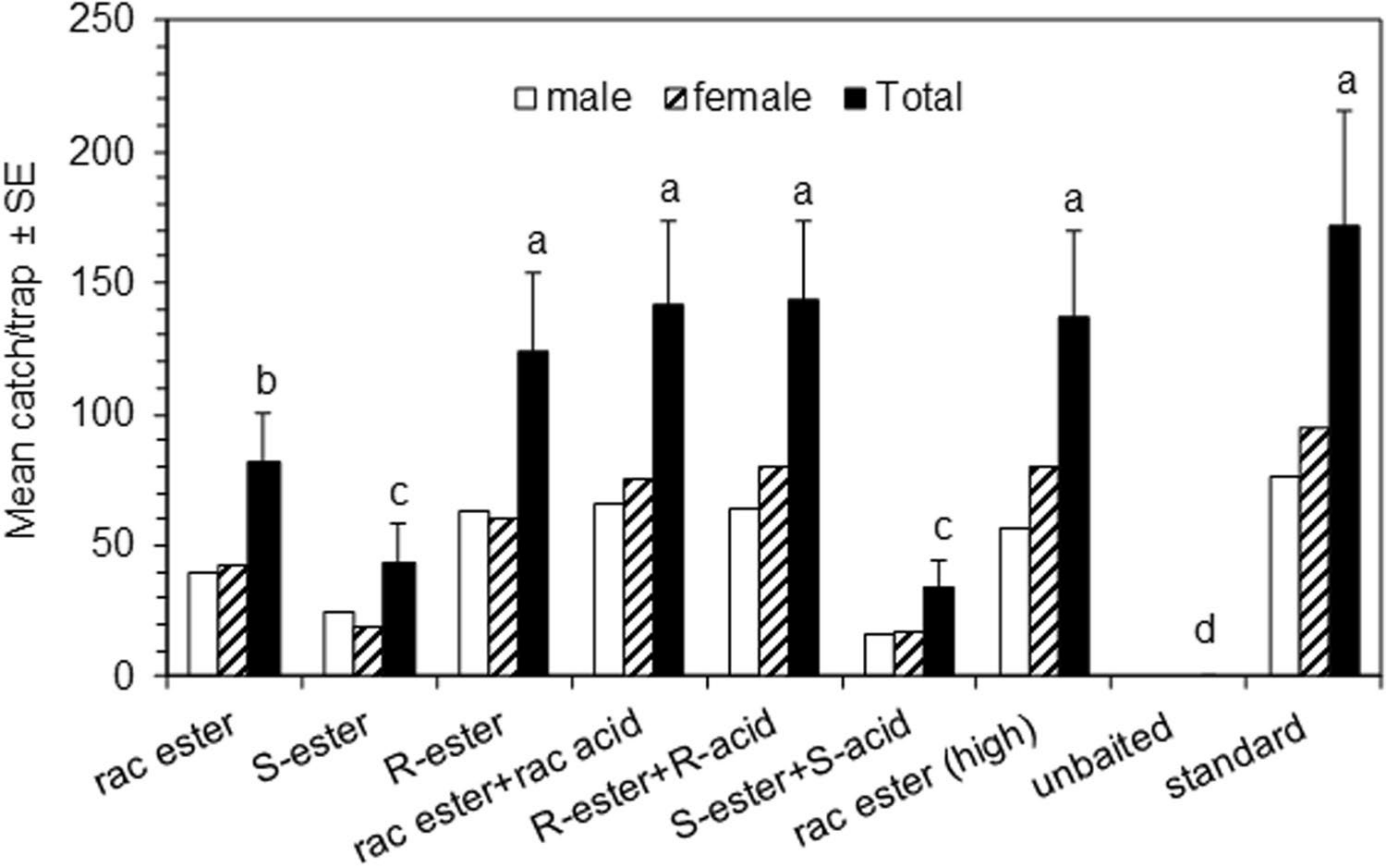
Mean catches of Guam strain *Oryctes rhinoceros* (CRB-G) beetles in traps in Experiment 2 (Solomon Islands; 20 June – 11 July 2017; *N* = 6). Means with different letters are significantly different (*P* < 0.05) after analysis of variance on total catches transformed to log(x + 1) and LSD test of significance of differences between means (*F* = 14.96; df = 7,35; *P* < 0.001). Ester is ethyl 4-methyloctanoate; acid is 4-methyloctanoic acid; standard is Sime Darby/ChemTica lure

In the second experiment (Fig. 6), addition of the corresponding acid to racemic ethyl 4-methyloctanoate significantly increased catches, although addition of the corresponding acids to the individual enantiomers of ethyl 4-methyloctanoate did not significantly affect catches. As in the first experiment, catches in traps baited with ethyl (*S*)-4-methyloctanoate and the combination with the corresponding acid were significantly lower than those in traps baited with the racemic compounds or (*R*)-enantiomers. Increasing the release rate of racemic ethyl 4-methyl alone by using a polyethylene sachet rather than the polyethylene vial increased catches to a level not significantly different from catches in traps baited with the ChemTica lure (release rates under laboratory conditions 1.0 mg/d, 20.0 mg/d and 9.1 mg/d, respectively; Table 1).

## Discussion

### Pheromone Components

The results described here indicate that ethyl 4-methyloctanoate is a component of the male-produced aggregation pheromone of the new haplotype Guam strain of *O. rhinoceros* (CRB-G), causing a strong EAG response from antennae of both male and female beetles and being attractive to both male and female beetles in field trapping tests. As such it may serve as a sex-aggregation pheromone (Cardé 2014), but this was not investigated further. The male beetles also produce 4-methyloctanoic acid in very variable amounts relative to the ethyl ester and sometimes the acid was produced with none of the ester detectable. The acid did not elicit an EAG response from male or female beetles. In field trapping tests, catches in traps baited with the acid were low, although significantly greater than those in unbaited traps which trapped no beetles, possibly due to the relatively restricted trap entrance. In most cases, addition of the acid to the ester did not affect catches, and only in one case it did significantly increase catches, so it seems unlikely that 4-methyloctanoic acid is an essential component of the aggregation pheromone. Similar results were reported previously for the standard strain of *O. rhinoceros* (CRB) by both Hallett et al. (1995) and Morin et al. (1996).

Ethyl 4-methyloctanoate is produced by males of all the other species of *Oryctes* reported to date: *O. agamemnon* (Said et al. 2015), *O. elegans* (Rochat et al. 2004), and *O. monoceros* (Allou et al. 2006; Gries et al. 1994) and attractive to both female and male conspecifics. In all species investigated, the corresponding acid was also reported to be produced, and in *O. elegans* it was the predominant component and more attractive to conspecifics than the ethyl ester, particularly when combined with date palm volatiles (Rochat et al. 2004). Methyl 4-methyloctanoate was reported in volatiles collected from *O. elegans* (Rochat et al. 2004), as were 4-methyloctan-1-ol and the corresponding acetate in volatiles from *O. agamemnon* (Said et al. 2015) and *O. elegans* (Rochat et al. 2004), but no behavioral function was found for these compounds. We could not detect the latter three compounds in volatiles from males of CRB-G or CRB-S. In EAG studies, the antennae of both male and female CRB-G gave strong and consistent responses to ethyl 4-methyloctanoate, occasional weak responses to methyl 4-methyloctanoate and no responses to 4-methyloctan-1-ol and the corresponding acetate.

Significant amounts of (*E*)-geranic acid were detected at one stage in collections from both male and female CRB-G beetles on sugarcane. It was thought this might have come from the sugarcane, but it was not possible to test this as that batch of sugarcane had been used up. Traps baited with (*E*)-geranic acid caught very low numbers of CRB-G beetles, although significantly more than the zero catches in unbaited traps.

### Stereochemistry

The stereochemistries of ethyl 4-methyloctanoate and 4-methyloctanoic acid produced by male CRB-G were determined by analysis on a commercially available β-cyclodextrin GC column. This gave baseline separation of the enantiomers of the acid, but no detectable separation of the enantiomers of the ester. However, the ester could easily be hydrolyzed to the corresponding acid on sub-microgram scale and its stereochemistry determined. Separation of the enantiomers of various 2-, 3- and 4-methylbranched acids by GC analysis on a modified γ-cyclodextrin phase was reported by Karl et al. (1994b). Heinsman et al. (1998, 2001) described separation of enantiomers of both 4-methyloctanoic acid and the corresponding ethyl and butyl esters on β-cyclodextrin phases. It is not known why we obtained no detectable separation of the esters on a similar phase; the only obvious difference is that we used helium as carrier gas while Heinsman et al. (1998, 2001) used hydrogen.

Using this approach, both ethyl 4-methyloctanoate and 4-methyloctanoic acid produced by male CRB-G beetles were determined to be exclusively the (*R*)-enantiomers. This was in contrast to the conclusion of Hallett et al. (1995) that male *O. rhinoceros* beetles from Indonesia produced the (*S*)-enantiomers, based on significantly higher catches of beetles in traps baited with the (*S*)-enantiomer than in those baited with the (*R*)-enantiomer. These authors were unable to determine the stereochemistries of the compounds produced by the beetles (Hallett et al. 1995). However, our analyses of ethyl 4-methyloctanoate and 4-methyloctanoic acid produced by CRB-S from Papua New Guinea showed that these were both the (*R*)-enantiomers, identical to those produced by CRB-G. Moreover, in our field trapping tests, traps baited with ethyl (*R*)-4-methyloctanoate caught more CRB-G than those baited with the (*S*)-enantiomer. Francke and Akasaka (pers comm 2018) also found CRB produced the (*R*)-enantiomer of ethyl 4-methyloctanoate by HPLC analysis of the fluorescent (*S*)- and (*R*)-1-(anthracene-2,3-dicarboximido)-2-propyl derivatives of the corresponding acid (cf. Mori and Akasaka, 2016).

The reasons for the different result of Hallett et al. (1995) with CRB from Indonesia can only be speculated on after over 25 years. The enantiomers of ethyl 4-methyloctanoate used by Hallett et al. (1995) were synthesized from enantiomers of citronellol. (*S*)-Citronellol (97.5% ee) gave ethyl (*R*)-4-methyloctanoate and (*R*)-citronellol (96% ee) gave the (*S*)-enantiomer. The stereochemistries of the enantiomers of ethyl 4-methyloctanoate produced could not be determined directly but the procedures used would have been expected to retain the stereochemistries and enantiomeric purities of the corresponding starting materials, i.e. similar to those tested in our work. Catches in the trapping experiment by Hallett et al. (1995) were lower than in our work (means <7 beetles per trap over two weeks) and differences in catches with the two enantiomers were numerically not great, even though statistically significant.

According to Marshall et al. (2017) both CRB-G and CRB-S were present in Indonesia at the time of their collections, but this was over 20 years later than the collections of Hallett et al. (1995), and one has to accept that it is entirely possible that the CRB population then inhabiting Indonesia produced and responded to the (*S*)-enantiomer of ethyl 4-methyloctanoate and the populations that now inhabit the recently-invaded islands produce and respond to the (*R*)-enantiomer of the same compound. Certainly more geographically widespread investigations of the stereochemistry of the pheromones produced by *O. rhinoceros* should be carried out to establish the current situation.

### Synthesis

The enantiomers of 4-methyloctanoic acid have previously been prepared by multistep syntheses from the enantiomers of citronellol (Hallett et al. 1995) or citronellal (Mori and Akasaka 2016) which are both commercially-available in high enantiomeric purities. Karl et al. (1994a) synthesized enantiomers of 2-methyl acids by chromatographic separation of the diastereoisomeric phenylglycinol amides followed by chain elongation to give both 3- and 4-methyl acids. Muñoz et al. (2009) used (*R,R*)- and (*S,S*)-pseudoephedrine-propionamides as chiral auxiliaries. Stereospecific alkylation with a protected 3-iodo-1-propanol was followed by removal of the auxiliary by hydrolysis, chain extension and oxidation.

In our work, the enantiomers of 4-methyloctanoic acid and the corresponding ethyl ester were conveniently resolved enzymatically in multigram quantities as reference standards and for field testing. Heinsman et al. (1998, 2001) reported that immobilized lipase from *Candida antarctica* acts selectively on the (*R*)-enantiomer*,* so that the acid can be selectively esterified in ethanol or the ethyl ester can be selectively hydrolyzed in aqueous buffer. Moreover, the acid could be separated from the ester by extraction with aqueous base and subsequent acidification without resort to chromatography. The stereoselectivity of this lipase for the enantiomers of 4-methyloctanoic acid was not as high as for 2-alkanols and the corresponding acetates (Hall et al. 2012), but two cycles produced material with at least 94% ee.

### Pheromone Production

In our hands, beetles of both CRB-G and CRB-S strains did not start producing pheromone for several months after emergence and arrival at NRI. In attempts to induce pheromone production, various substrates including palm detritus, soil and paper were tried, as were various foodstuffs including banana, apple, palm hearts and sugarcane, without any obvious effects. Beetles were kept under laboratory conditions and did not fly, so whether this pattern is replicated in the field is unknown. 4-Methyloctanoic acid may be a biosynthetic precursor of the corresponding ethyl ester and conversion of this to the ester may require ethanol from microbial activity in the detritus typically inhabited by *O. rhinoceros*. Microbial activity was kept to a minimum under laboratory conditions which might have delayed esterification of the acid, but this would not explain the delayed production of 4-methyloctanoic acid. This phenomenon has not been reported for other *Oryctes* species, although Prof Didier Rochat did suggest that pheromone production can be very variable and erratic (pers comm 2018).

### Practical Aspects

Ethyl 4-methyloctanoate is highly attractive to both CRB-G and CRB-S strains of *O. rhinoceros.* Male beetles produce the (*R*)-enantiomer, and this enantiomer was most attractive in the field tests. The (*S*)-enantiomer was significantly less attractive in both tests, although it is not possible to say whether the (*S*)-enantiomer is attractive at all as the material used in our field tests contained small amounts of the (*R*)-enantiomer. The racemic material was just as attractive as the (*R*)-enantiomer in the first field test, although less attractive in the second test. However, it should be noted that the same dispensing devices were used for the racemic compound and the separate enantiomers, such that the release rate of the (*R*)-enantiomer from dispensers containing the racemic compound would have been half that from dispensers containing the pure enantiomer.

Catches of CRB-G beetles with racemic ethyl 4-methyloctanoate released at 1 mg/d were generally less than those with the standard ChemTica lure releasing at 9.1 mg/d under laboratory conditions, although not significantly so in the first field trapping experiment. Increasing the release rate to 20.0 mg/d significantly increased catches relative to catches with a release rate of 1 mg/d in the second experiment, although not significantly more than catches with the standard dispenser at 9.1 mg/d.

As indicated above, catches of CRB-G in traps baited with racemic 4-methyloctanoic acid or the individual enantiomers were low. Addition of the racemic acid at 1.4 mg/d to racemic ethyl 4-methyloctanoate at 1.0 mg/d significantly increased catches in the second experiment, although addition of the individual enantiomers of the acid to the corresponding ethyl ester had no significant effect in either case. Thus, although (*R*)-4-methyloctanoic acid is probably not an essential component of the naturally-produced aggregation pheromone which seems to consist of the single component, ethyl (*R*)-4-methyloctanoate, it may be possible to increase the attractiveness of the more readily available racemic ester by addition of the racemic acid. Depending upon the costs of production of the compounds and the manufacture of two separate lures, it may be economically advantageous to add the racemic acid released at 1.4 mg/d to the racemic ethyl 4-methyloctanoate released at 1 mg/d rather than using one lure releasing racemic ethyl 4-methyloctanoate at 9.1 mg/d.

## Conclusions

The aim of this work was to compare the compositions of the aggregation pheromones produced by male *O. rhinoceros* beetles of the two haplotypes reported by Marshall et al. (2017), CRB-G and CRB-S, and to evaluate the attractiveness of lures containing the synthetic pheromone in the field. Males of both haplotypes produce ethyl (*R*)-4-methyloctanoate and the corresponding acid with no consistent qualitative or quantitative differences. Only the former compound elicited electrophysiological responses from receptors on the antennae of female and male beetles. Field trapping tests carried out in an area where the CRB-G haplotype was reported to predominate showed that ethyl (*R*)-4-methyloctanoate was more attractive to male and female beetles than the (*S*)-enantiomer. However, the racemic compound, used in commercial lures, was equally attractive, and the current commercial lures are highly attractive to the CRB-G haplotype of this pest.

Very recently, Etebari et al. (2021) reported that the occurrence of mitochondrial haplotypes and association with resistance to OrNV infection may be more complex than the binary system reported by Marshall et al. (2017) and Reil et al. (2018). Etebari et al. (2021) found three major mitochondrial haplotype groups across the South Pacific region and a high incidence of OrNV infection was detected in all haplotypes wherever they occurred. They suggested that the current method, based on the sequence of the mitochondrial *CoxI* gene, is not a reliable diagnostic marker for phenotypic traits, and further molecular analyses will be required to identify possible mechanisms of resistance to OrNV, combined with an improved understanding of the population genetics of the pest and the evolutionary history of OrNV in the region (Etebari et al. 2021). While re-evaluation of our work here may be required in the light of such findings, demonstration that *O. rhinoceros* beetles from a population reliably reported to be resistant to OrNV produce and respond to the same pheromone produced and responded to by insects from a susceptible population remains valid.

## Supplementary Information

ESM 1 (PDF 438 kb)
