# Supplementary material for: Identification of Components of the Aggregation Pheromone of the Guam Strain of Coconut Rhinoceros Beetle, Oryctes rhinoceros, and Determination of Stereochemistry
Source: J Chem Ecol. 2021 Nov 11;48(3):289–301. doi: 10.1007/s10886-021-01329-z (PMC8934761; doi:10.1007/s10886-021-01329-z)
Supplement: Supplementary file 1 — (PDF 438 kb) [file 10886_2021_1329_MOESM1_ESM.pdf]

Identification of Components of the Aggregation Pheromone of the Guam Strain of Coconut Rhinoceros Beetle, *Oryctes rhinoceros*, and Determination of Stereochemistry

David R Hall[1] Steven J Harte[1] Dudley I Farman[1] Mark Ero[2,4] Alfred Pokana[3]

[1] Natural Resources Institute, University of Greenwich, Central Avenue, Chatham Maritime, Kent ME4 4TB, UK

[2] Papua New Guinea Oil Palm Research Association, Dami Research Station, PO Box 97 Kimbe, West New Britain Province, Papua New Guinea

[3] New Britain Palm Oil Limited, Quadalcanal Plains, PO Box 2001, Honiara, Solomon Islands

[4] Current address: Pacific Community (SPC), Land Resources Division (LRD), Private Mail Bag, Suva, Fiji

Supplementary Material

Resolution of 4-Methyloctanoic Acid

4-Methyloctanoic acid (15.8 g, 100mM; SigmaAldrich, Gillingham, Kent) was dissolved in absolute ethanol (100 ml) and stirred with immobilized lipase from *Candida antarctica* (200 mg; SigmaAldrich) at room temperature. Progression of the reaction is shown in Table S1.

After 72 h, the suspension was filtered through cotton wool and the ethanol was removed on a rotary evaporator. The residue was dissolved in 10% diethyl ether in petroleum spirit (bp 40-60°C) (100 ml) and extracted twice with water (20 ml) and the water re-extracted with 10% diethyl ether in petroleum spirit (bp 40-60°C) (100 ml). The organic solutions were then extracted twice with 2N KOH solution (50 ml), and the combined aqueous extracts extracted again with 10% diethyl ether in petroleum spirit (bp 40-60°C) (50 ml). The combined organic extracts were extracted once with saturated NaCl solution and dried with magnesium sulphate. The combined aqueous extracts were acidified with 4N sulfuric acid (30 ml), saturated with

sodium chloride and extracted three times with diethyl ether (50 ml). The combined organic extracts were washed once with brine (50 ml) and dried over magnesium sulfate.

Table S1. Time course of esterification of 4-methyloctanoic acid catalyzed by lipase from *Candida antarctica* showing relative amounts of ethyl 4-methyloctanoate (ester) and the remaining amounts of the (*R*)- and (*S*)-enantiomers of 4-methyloctanoic acid (acid)

	Relative amounts (%)		
Time (h)	Ester	(*R*)-acid	(*S*)-acid
0.5	1.2		
2.5	8.5	36.1	45.3
18	36.5	13.9	40.8
21	40.1	11.0	40.5
24	41.2	9.3	40.5
26	44.2	8.0	39.6
43	50.4	2.8	34.7
48	51.9	2.6	37.5
65	53.9	1.4	37.5
72	54.7	1.1	37.5

The acidic extract was filtered through silica gel (2 gm) and the solvent removed on a rotary evaporator to give the **(*S*)-acid (8.3 g; R/S 2.2/83.0; 94.7% ee).** Of this, 4.3 g was kugelrohr distilled (160°C/10 mm) to give 3.8 g (*S*)-acid.

The remaining 4 g of (*S*)-acid was dissolved in absolute ethanol (40 ml) and boron trifluoride etherate (10 drops) added (65 h 89%, 89 h 93%, 138 h 96%, 7 d 99%). After stirring for 7 d at room temperature the ethanol was removed on a rotary evaporator, the residue dissolved in petroleum spirit (50 ml), washed with 20 ml portions of water, 2N KOH, water, brine, dried over magnesium sulfate. Removal of solvent on a rotary evaporator and kugelrohr distillation (120°C/10 mm) gave the **S-ester (4.0 g).**

Removal of solvents from the ester fraction above gave 10.7 g. A small sample was hydrolyzed in 1:1 ethanol/2N KOH overnight to give the acid for analysis of the enantiomeric composition on the cyclodextrin GC column (R/S 89:11). To this ester was added 1M K_2HPO_4 (60 ml) and the immobilized enzyme used in the previous reaction, and the mixture stirred at room temperature (ester/R-acid/S-acid after 1 h 51.6/39.4/2.6; 1.5 h 37.4/55.1/2.5; 2h 33.3/59.4/2.8). After 2 h the mixture was filtered through Celite and subjected to above workup (2 x 25 ml 2N KOH, 15 ml 4N H_2SO_4). The acidic fraction was filtered through silica gel (2 g) and solvents removed to give 5.7 g. Of this, 2.7 g was kugelrohr distilled (160°C/10 mm) to give the R-acid (2.4 g 92% ee).

The remaining acid (3.0 g) was dissolved in ethanol (20 ml) and stirred with immobilized enzyme (76 mg) at room temperature (1 h 11.8/74.3; 4 h 33.5/50.3; 6 h 42/34; 48 h 85.1/3.2/1.7). After 48 h, aqueous workup and kugelrohr distillation gave the **R-ester (3. 0 g; trace S approx. 98% ee)**.

To the ester fraction (3.5 g; R/S 75.9/17.8) was added 0.5 M K_2HPO_4 (20 ml) and the same immobilized enzyme as above (1.5 h 43.8/31.3/1.5; 3 h 38.3/45.0/3.0; 5 h 36.3/49.6/4.1; 6 h 33.3/49.6/4.5). After 6 h, work up gave the ester fraction (1.25 g R/S 60.4:33.6) and acidic fraction 1.8 g (R/S 88.3:11.7). This was dissolved in ethanol (12 ml) and stirred with the same immobilized enzyme at room temperature for 24 h. Workup and kugelrohr distillation gave the **R-ester (1.1 g, R/S 90.4:1.8; 96.2% ee)** and the acid fraction (0.4 g; R:S 73.9:23.7).

Other Syntheses

Ethyl 4-methyloctanoate 4-Methyloctanoic acid (25 gm, 0.158M; SigmaAldrich, Gillingham, Kent, UK) was dissolved in ethanol (300 ml) and 2 drops of 2N sulfuric acid in a 500 ml-round-bottomed flask. The solution was refluxed for 1 h, after which most of the ethanol was distilled off at atmospheric pressure. A further portion of ethanol (300 ml) is added and the procedure repeated twice more until the reaction was complete by GC and TLC. The residue was dissolved in petroleum spirit (bp 60-80°C, 100 ml) and washed with 5% sodium hydroxide solution and brine, back extracting the aqueous extracts with petroleum spirit (100 ml). The organic extracts were dried with magnesium sulphate, filtered and the solvent removed on a rotary evaporator.

The residue was vacuum distilled giving a main fraction (bp 98°C/8 mm, 25.2 gm, 86%); mass spectrum Fig. S1; ^{1}H and ^{13}C NMR spectra in agreement with Mori et al. (2016).

Methyl 4-methyloctanoate 4-Methyloctanoic acid (0.32 mg; 2 mM) was dissolved in methanol (5 ml) and one drop of borontrifluoride etherate added. After 5 d most of the methanol was removed on a rotary evaporator and aqueous workup and kugelrohr distillation (150 ºC/20 mm Hg) gave methyl 4-methyloctanoate (0.28 g; 82%); mass spectrum Fig. S2; ^{1}H NMR (400 MHz, $CDCl_3$) δ ppm: 3.67 (s, 3H); 2.25-2.4 (m, 2H), 1.6-1.7 (m, 1H), 1.35-1.5 (m, 2H), 1.2-1.3 (m, 5H), 1.1-1.2 (m, 1H), 0.88 (d and t, 6H); ^{13}C NMR (101MHz, $CDCl_3$) δ ppm: 174.65, 51.50, 36.32, 32.36, 31.89, 29.13, 22.95, 19.28, 14.13.

4-Methyl-1-octanol Ethyl 4-methyloctanoate (0.93 g; 5 mM) was dissolved in dry ether (10 ml) cooled in ice and reacted with lithium aluminum hydride (0.19 g; 5 mM). After 1 h the mixture was treated successively with water (0.2 ml), 2N KOH (0.2 ml) and water (1 ml), filtered, dried with anhydrous magnesium sulfate and kugelrohr distilled (140 ºC/15 mm Hg) to give 4-methyl-1-octanol (0.63 g; 89%); mass spectrum Fig. S2; ^{1}H and ^{13}C NMR spectra in agreement with Mori et al. (2016).

4-Methyl-1-octyl acetate 4-Methyl-1-octanol (0.29 g; 2 mM) was dissolved in dry pyridine (0.3 ml) and acetic anhydride (0.3 ml) added. After 4 h, aqueous workup and kugelrohr distillation gave 4-methyl-1-octyl acetate (0.32 g; 80%); mass spectrum Fig. S2; ^{1}H NMR (400 MHz, $CDCl_3$) δ ppm: 4.04 (t, J = 6.8 Hz, 2H), 2.05 (s, 3H), 1.5-1.7 (m, 2H), 1.1-1.7 (m, 9H), 0.88 (d and t, 6H); ^{13}C NMR (101MHz, $CDCl_3$) δ ppm: 171.29, 65.01, 36.57, 33.03, 32.45, 29.25, 26.16, 23.00, 21.05, 19.56, 14.16.

Table S2 Retention indices of compounds relative to retention times of *n*-alkanes on non-polar HP5 and polar DBWax GC columns

	Retention Index	
Compound	HP5	DBWax
Ethyl 4-methyloctanoate	1256	1463
4-methyloctanoic acid	1235	2083
Methyl 4-methyloctanoate	1184	1418
4-Methyl-1-octanol	1135	1588
4-Methyl-1-octyl acetate	1271	1563
(*Z*)-Geranic acid	1322	2254
(*E*)-Geranic acid	1398	2286

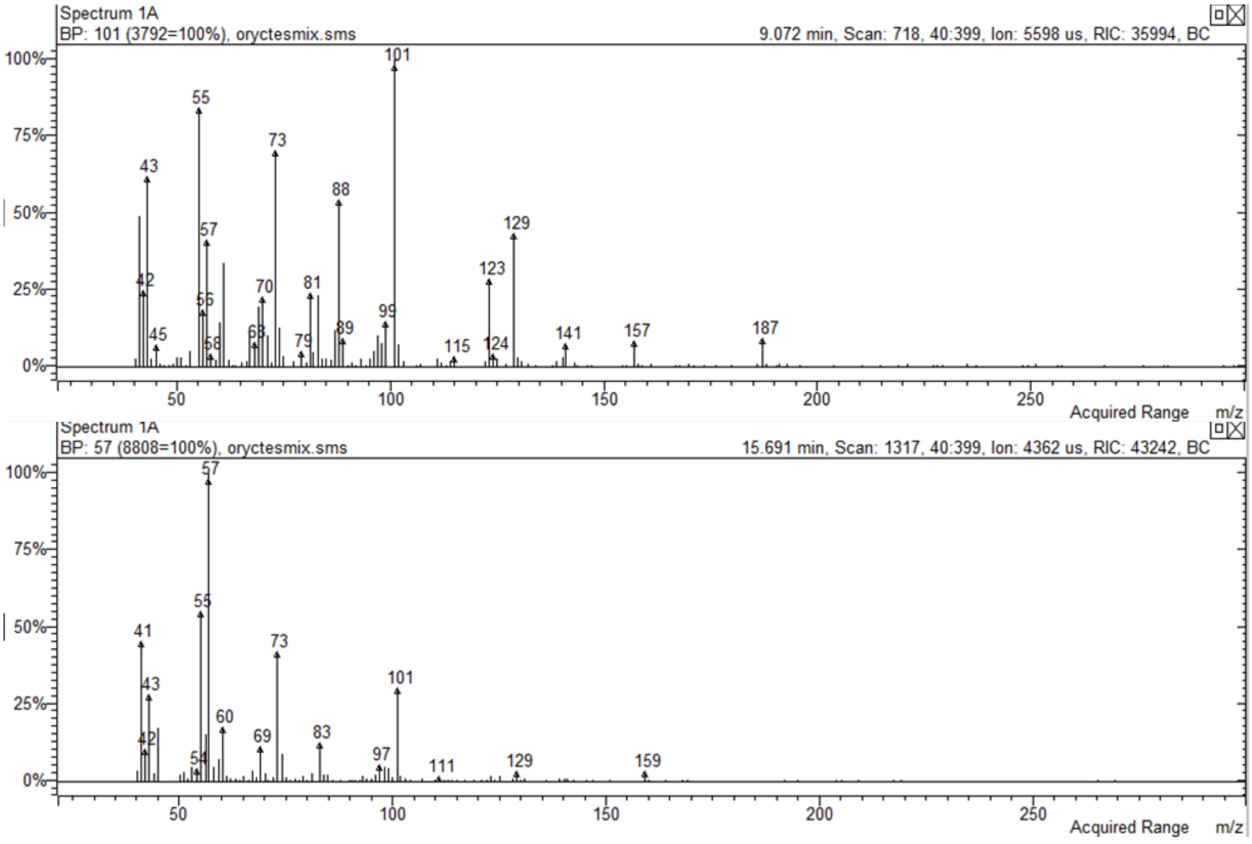

Fig. S1 Mass spectra of ethyl 4-methyloctanoate (upper) and 4-methyloctanoic acid (lower)

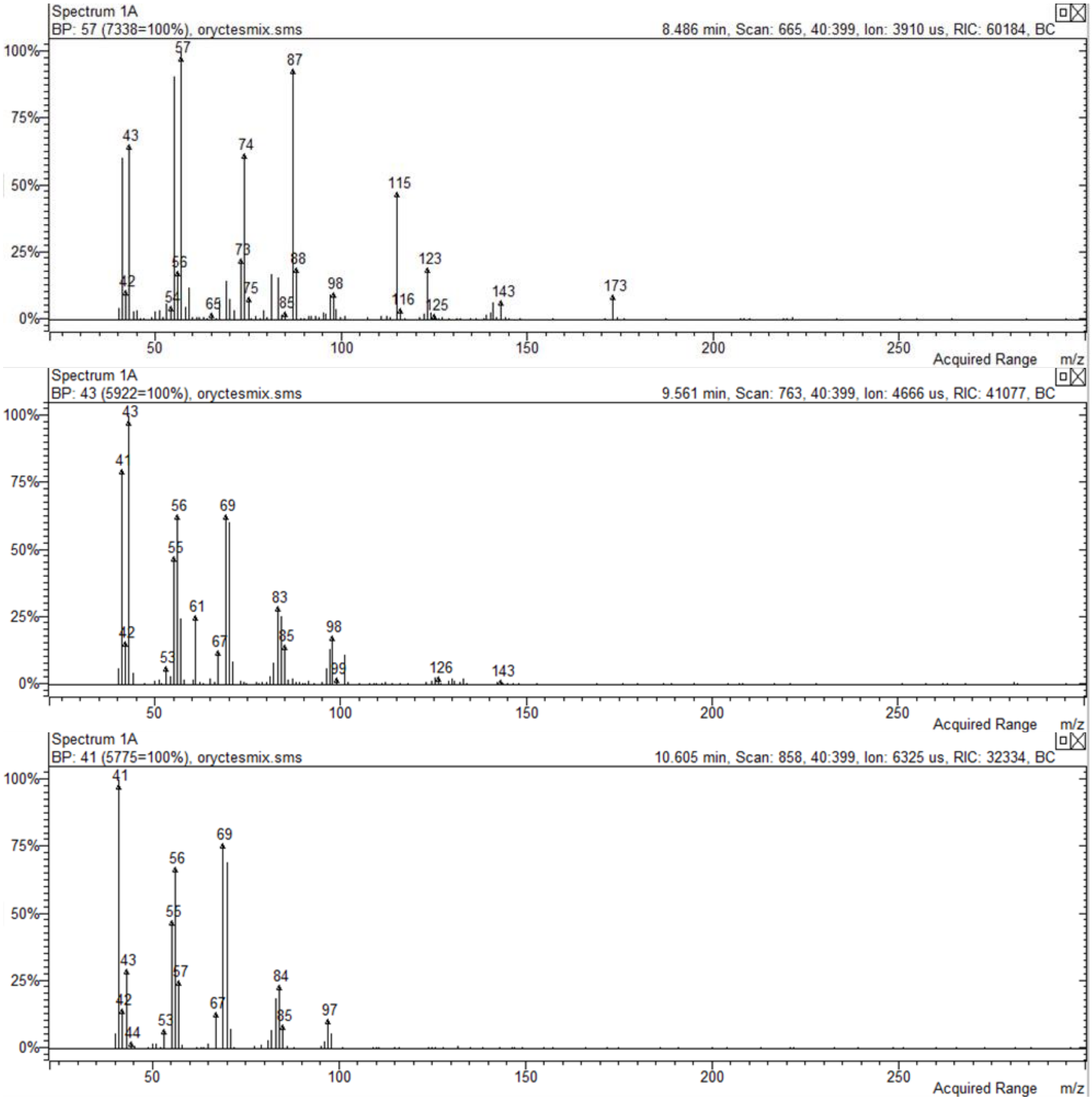

Fig. S2 Mass spectra of (from top) methyl 4-methyloctanoate, 4-methyl-1-octyl acetate, 4-methyl-1-octanol

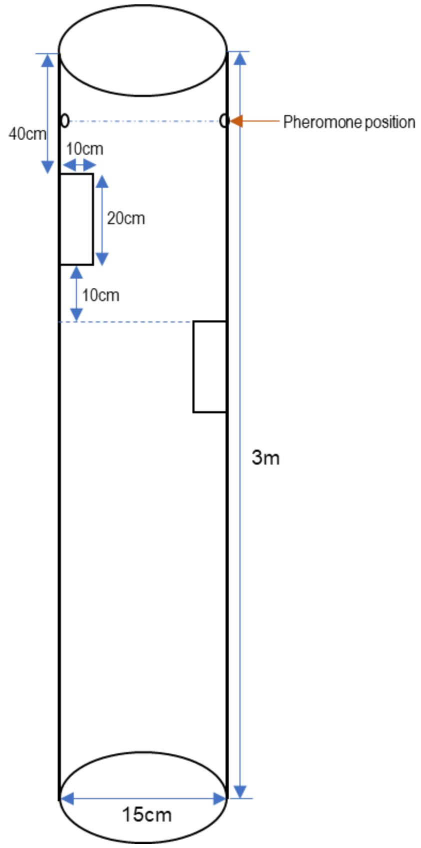

Fig. S3 Diagram of trap used in field trapping experiments in Solomon Islands

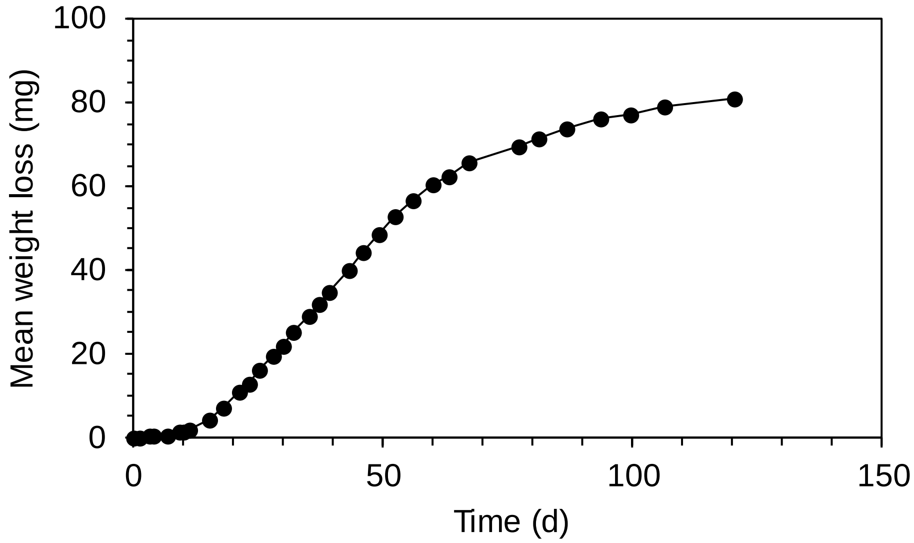

Fig. S4 Release of racemic ethyl 4-methyloctanoate from polyethylene vial (30 mm x 15 mm x 1.5 mm thick) at 20-22 ºC as measured by weight loss (*N* = 2)

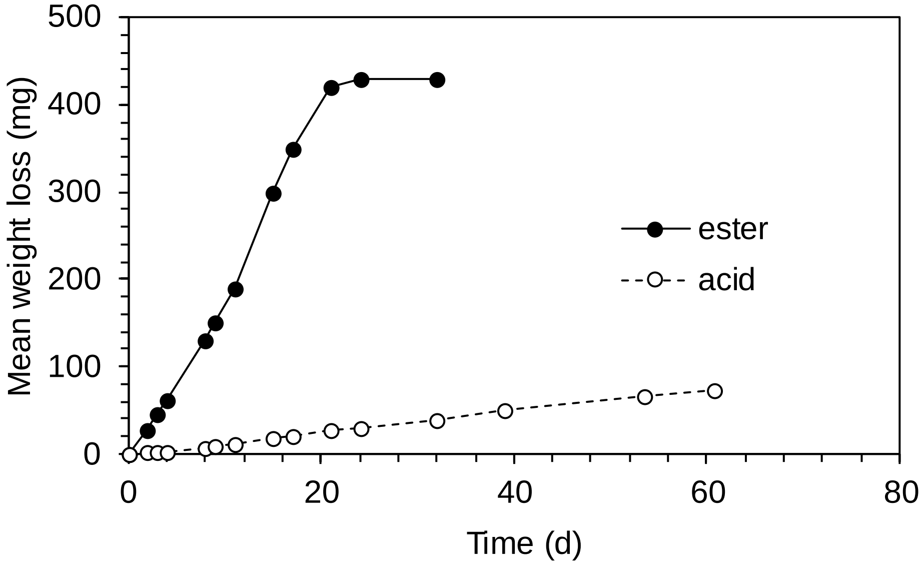

Fig. S5 Release of racemic ethyl 4-methyloctanoate (ester) and 4-methyloctanoic acid (acid) from polyethylene sachets (2.5 cm x 5 cm x 250 µm) at 20-22 ºC, as measured by weight loss (*N* = 2)

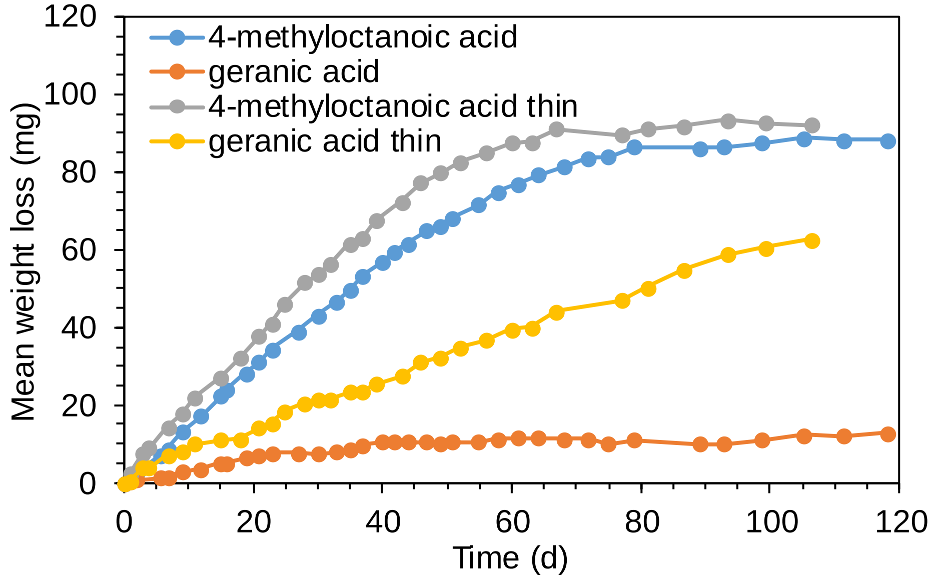

Fig. S6 Release of 4-methyloctanoic acid and (*E*)-geranic acid from polyethylene sachets (5 cm x 5 cm x 120 µ thick; thin 5 cm x 5 cm x 60 µm thick) at 20-22 ºC as measured by weight loss (*N* = 2)

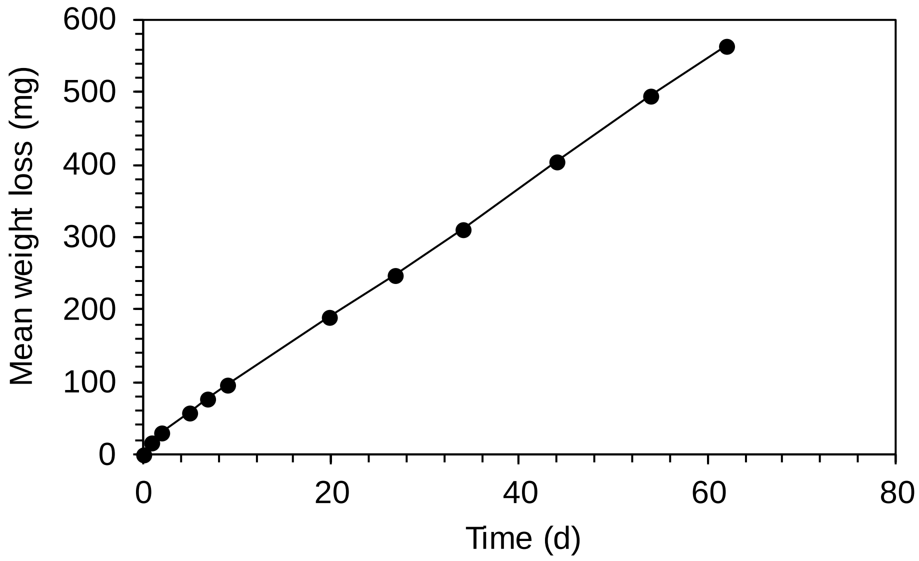

Fig. S7 Release of ethyl 4-methyloctanoate from Sime Darby/ChemTica dispenser at 20-22 ºC as measured by weight loss (*N* = 2)

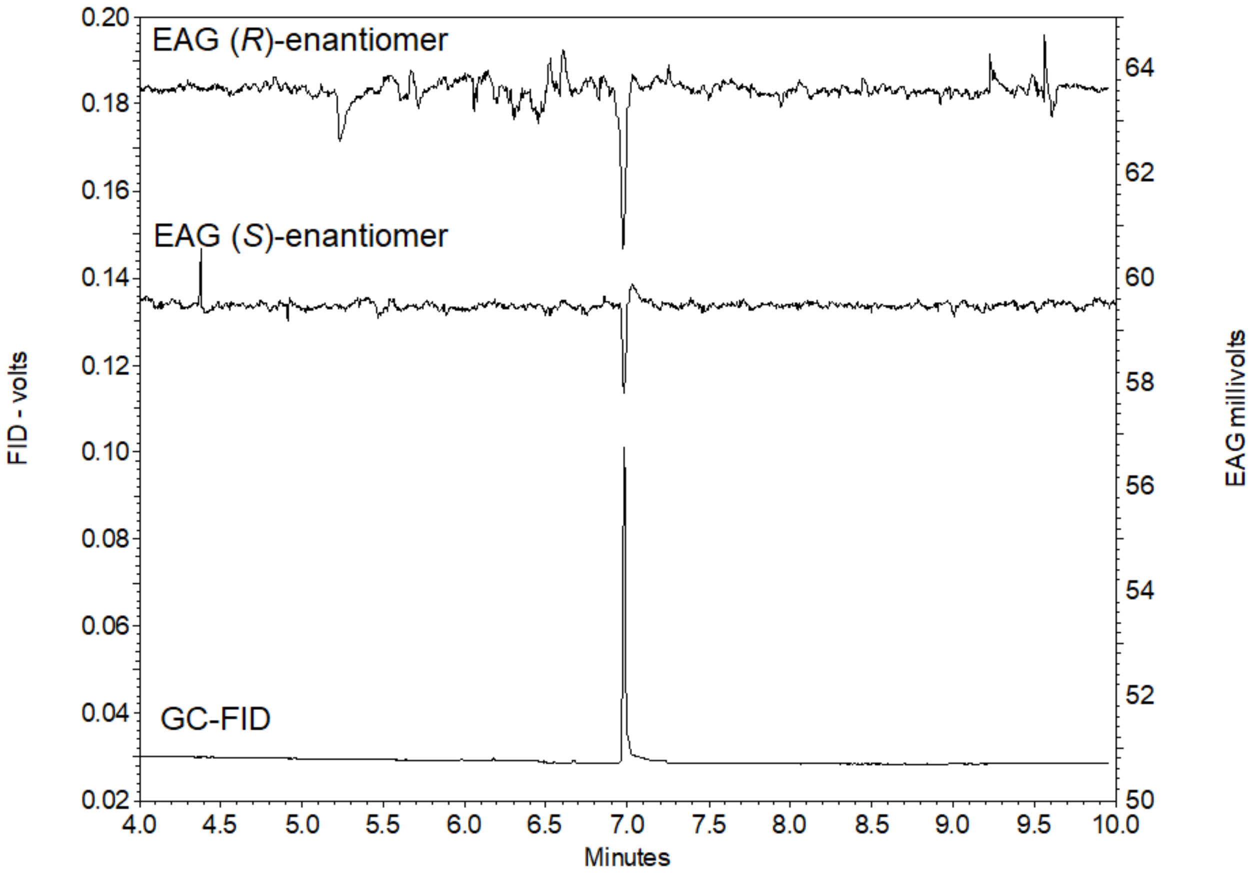

Fig. S8 Representative GC-EAG analyses of ethyl (*R*)-4-methyloctanoate (upper) and the (*S*)-enantiomer with antenna of female CRB-G

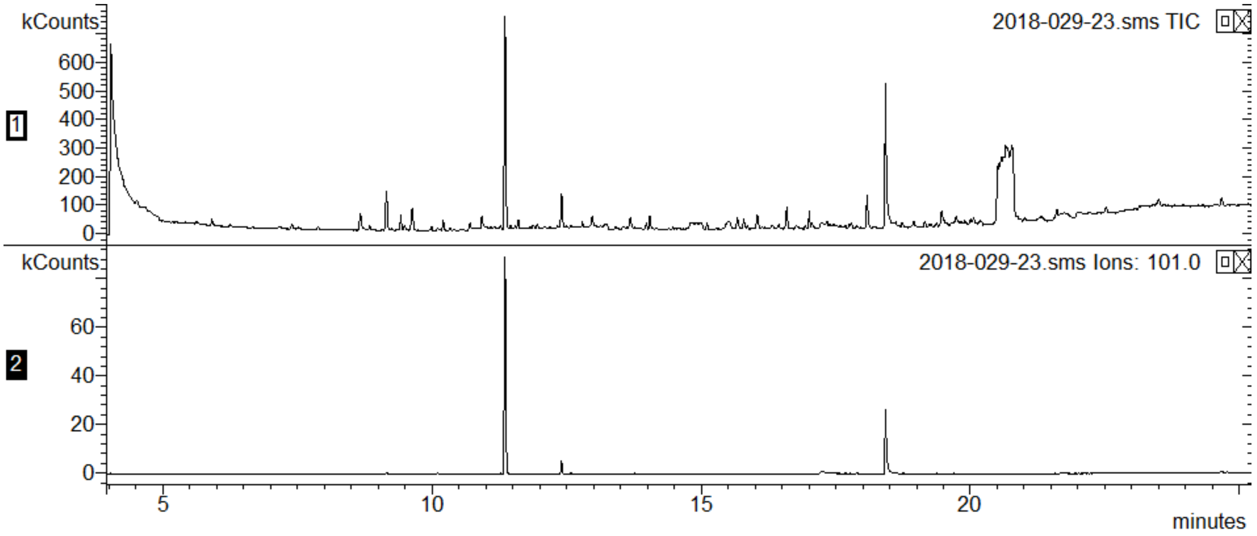

Fig. S9 GC-MS Chromatogram on polar GC column of collection of volatiles from male susceptible strain *Oryctes rhinoceros* (CRB-S): upper trace TIC, lower trace single ion monitoring at *m/z* 101, showing ethyl 4-methyloctanoate at 11.36 min and 4-methyloctanoic acid at 18.42 min

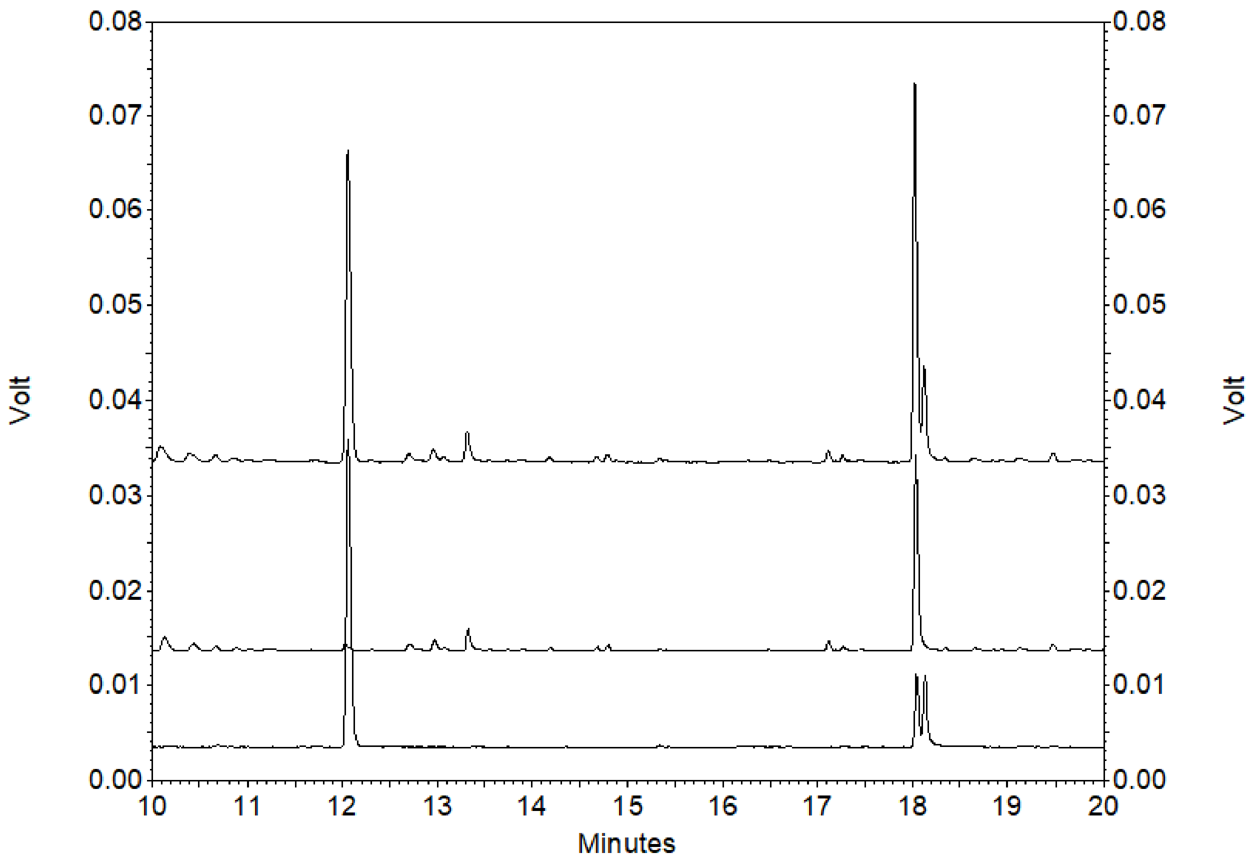

Fig. S10 GC Analyses on cyclodextrin column of (from bottom) racemic ethyl 4-methyloctanoate (12.10 min) and racemic 4-methyloctanoic acid (*R* 18.06 min and *S* 18.14 min), (*R*)-4-methyloctanoic acid after hydrolysis of collection of volatiles from standard strain of *Oryctes rhinoceros* (CRB-S), and co-injection of racemic standards with hydrolyzed collection

Reference

Mori K, Akasaka K (2016) Pheromone synthesis. Part 258. Synthesis of the enantiomers of the beetle pheromones ethyl 4-methylheptanoate, 4-methyloctanoic acid and 4-methyl-1-nonanol, and HPLC analysis of their derivatives to determine their enantiomeric purities. Tetrahedron-Asymmetr 27:182–187
